# Impact of Ultrasonication on African Oil Bean (*Pentaclethra macrophylla* Benth) Protein Extraction and Properties

**DOI:** 10.3390/foods12081627

**Published:** 2023-04-12

**Authors:** Blessing C. Nwokocha, Afroditi Chatzifragkou, Colette C. Fagan

**Affiliations:** Department of Food and Nutritional Sciences, University of Reading, Whiteknights RG6 6DZ, UK

**Keywords:** African oil bean, ultrasonication, protein, properties, oilseed, functional properties, legumes, Sub-Saharan

## Abstract

African oil bean (Pentaclethra macrophylla Benth) is an underutilised edible oil seed that could represent a sustainable protein source. In this study, the impact of ultrasonication on the extraction efficiency and properties of protein from African oil bean (AOB) seeds was evaluated. The increase in the duration of extraction favoured the extraction of AOB proteins. This was observed by an increase in extraction yield from 24% to 42% (*w*/*w*) when the extraction time was increased from 15 min to 60 min. Desirable properties were observed in extracted AOB proteins; the amino acid profile of protein isolates revealed higher ratios of hydrophobic to hydrophilic amino acids compared to those of the defatted seeds, suggesting alterations in their functional properties. This was also supported by the higher proportion of hydrophobic amino acids and high surface hydrophobicity index value (3813) in AOB protein isolates. The foaming capacity of AOB proteins was above 200%, with an average foaming stability of 92%. The results indicate that AOB protein isolates can be considered promising food ingredients and could help stimulate the growth of the food industry in tropical Sub-Saharan regions where AOB seeds thrive.

## 1. Introduction

Livestock production has served as a major source of protein through a wide range of meat products. The increase in demand and consumption of meat globally has resulted in detrimental impacts on the environment, such as an increase in methane emissions, which contribute to global warming, as well as increased water abstraction and resource depletion [1,2]. The food industry can play a significant role in addressing Sustainable Development Goals through the development of new, plant-based protein products, thereby helping to reduce the dependence on animal protein sources. This could assist in minimising the impact of protein consumption on the environment while, at the same time, providing the world’s growing population with the protein quantity needed [2]. The global population is on the increase, with a prediction of over 9.8 billion by 2050, which means that there will be more people to feed in the future [3]). This has resulted in a notable market increase in protein-rich food products. Therefore, there is an urgent need to diversify protein sources, and the exploitation of plant proteins is seen as a major solution.

African oil bean (*Pentaclethra macrophylla* Benth) is a leguminous tree with oil-bearing seeds. It grows wild without commercial cultivation in the humid lowlands of West and Central Africa and has no known varieties. It is valued in southeastern Nigeria for its soil improvement properties [4]. African oil beans (AOB) are edible oil seeds that are rich in protein (22%) and oil (52%) [5]. AOB is characterised by its bountiful harvest in Nigeria and other African countries [6]. Natural fermentation is the most practiced processing method of these oil seeds and is traditionally handed down from generation to generation. It is characterised by problems related to product safety, quality, and inconsistency, stemming from poor manufacturing practices and the absence of controlled processing parameters such as pH, temperature, humidity, and water quality [7]. Therefore, to improve the exploitation of AOB seeds, new utilisation strategies should be developed, which could lead to new value-added ingredients originating from AOB seeds.

Therefore, setting up industries for the manufacture of protein isolates from AOB seeds in Nigeria, particularly in the southeast region, would be cost-effective by eliminating the cost of transporting raw materials to high-income countries for production, as is seen in the case of Nigerian crude oil. Apart from being a source of nutrients, revenue and growth of the gross domestic product (GDP) of the nation, the exploitation of underutilised plant materials, such as AOB seeds, would stimulate the growth of the local food industry [8]. However, AOB is largely underutilised due to a lack of awareness of its nutritional benefits and inadequate storage and processing techniques. In addition, the annual wastage of these seeds due to postharvest losses, poor storage and processing facilities, and the predominant hunger challenge in the regions where these seeds are grown represent drivers for investigation [5].

In order to increase the exploitation of plant proteins, one major factor to consider is the extraction of proteins from plant materials [9]. The plant cell wall acts as a natural barrier for protein diffusion, and as such, techniques that improve extraction efficiency are desired. Various methods have been investigated, such as alkaline extraction, which adopts a simultaneous process of cell disruption and protein extraction, using aqueous NaOH, KOH and ammonia solutions [10] or organic solvents, which include ethanol and isopropanol [11]. Increased recovery yields can be achieved during alkaline extraction at elevated temperatures; however, extensive denaturation and protein aggregation may also occur, limiting protein quality and utilisation in food [12]. Ultrasonication technology has been extensively researched as a green technique for protein extraction from various plant-based materials, such as soybean flakes, peanut meal, rice bran, and legumes [13,14]. The application of ultrasonication in plant protein studies follows two distinctive but parallel paths: (i) the enhancement of protein extraction yields and subsequent protein modifications and (ii) the modification of the physicochemical properties of the protein [15]. Values on the increase in protein extraction yields from oilseed meals vary in the literature, ranging from 10% to 50% (*w*/*w*) [13,15,16]. With regard to the physical changes in proteins during ultrasonication, it has been highlighted that cavitation phenomena can unfold, denature, and reaggregate proteins. Moreover, ultrasonication causes modification in the protein structure. Specifically, low-intensity ultrasound (<1 W) treatments result in the partial unfolding of proteins and the consequent nontypical association of proteins to form aggregates because of the breakdown of non-covalent bonds as well as changes in the protein’s secondary and tertiary structures [17].

This study was aimed at investigating the effect of ultrasonication on the extraction efficiency of AOB seeds under alkaline conditions. Chemical characterisation of AOB protein was also carried out to investigate and provide insights on the impact of ultrasonication on AOB protein in terms of protein structure and functional properties.

## 2. Materials and Methods

### 2.1. Raw Material

Two batches of African oil bean (AOB) seeds were purchased from an open market in Abia state, Eastern Nigeria (November 2016—AOB1 and April 2018—AOB2). The seeds are flat and brown and are contained in a brown, flattened pod [18,19]. They have an average length of 56 mm and a width of 37 mm [5]. The number of seeds in each pod depends on the length of the pod and on the size of the seeds within (usually 6 to 10 seeds in each pod). The main method of harvesting is the manual collection of mature dispersed seeds from around the tree. The kernel, which is a dicotyledon, is embedded in a glossy brownish seed coat [18]. Both batches of AOB seeds were harvested, separated from the pods manually, and sold fresh without drying. Due to the hard shells, the seeds were intact and stored at 4 °C before analysis. Both batches were of similar appearance. The shells of AOB seeds were removed using a mechanical pressing machine that formed cracks on the hard shells and eased the complete removal by hand-peeling. A coffee grinder (DeLonghi, Treviso, Italy) was used to mill both seeds separately into a reduced particle size of approximately 0.75 mm diameter for analysis. Milled seeds were defatted using the Soxhlet method and were stored in a freezer at −20 °C prior to analysis.

### 2.2. Compositional Analysis of AOB Seeds

Moisture and ash were determined, in triplicate, as described in methods 934.01 and 942.05, respectively, in AOAC [20]. Total lipid content was determined gravimetrically using the Soxhlet extraction method and petroleum ether (Sigma-Aldrich, Gillingham, UK) as solvent. Crude protein was determined as described in method 990.03 in AOAC [20]. The structural carbohydrates of AOB seeds were determined by a two-step hydrolysis method as described by Sluiter et al. [21]. Briefly, 300 mg of defatted AOB sample was weighed in duplicate into test tubes and 3 mL of 72% (*w*/*w*) H_2_SO_4_ was added. Samples were placed in a water bath at 30 °C for 60 min. The mixture was diluted to 4% (*w*/*w*) H_2_SO_4_ by adding deionised water and treated at 121 °C in an autoclave for 60 min. The hydrolysates were cooled to room temperature and pH was increased to 5–6 using CaCO_3_ powder (Fisher scientific, Loughborough, UK). Supernatants were filtered with 0.2 μm filter size and analysed in an HPLC system (Agilent Infinity 1200 series, Didcot, UK), coupled with RI and DAD detectors. An Aminex 87H column (Biorad, Watford, UK) was used for sugar separation, at 65 °C and 0.005 M H_2_SO_4_ was used as mobile phase at a flow rate of 0.6 mL/min. Quantification of sugars was performed via calibration curves of external standards.

Acid-soluble lignin was determined by the absorbance of the hydrolysed liquor samples at 320 nm on a UV-visible spectrophotometer, using deionised water as blank. The acid-soluble lignin (ASL) was calculated according to Equation (1) below:

(1) $$\%\;\mathrm{ASL}=\left.\;\left(\frac{\left(\mathrm{UVabs}\right)\times \;\mathrm{Volume}\;\mathrm{filtrate}\;\times \;\mathrm{dilution}\;\mathrm{factor}\;}{\mathrm{ODWsample}\;\times \;\mathrm{Pathlength}}\right.\right)\times 100^{}$$

where ODW_sample_ is the dry weight of sample, UV_abs_ is average UV-Vis absorbance for the sample at 320 nm.

All compositional analyses were carried out in triplicate on both batches of seeds with the exception of acid-soluble lignin, which was carried out in duplicate.

### 2.3. Ultrasound-Assisted Protein Extraction of Defatted AOB Seeds

Defatted AOB seeds were subjected to ultrasound-assisted extraction as described by Dong et al. [22]. The ultrasonication process was carried out using an ultrasonic generator (model: P100/6-20, Sonic Systems Ltd., Puckington, UK) with a horn diameter of 3.5 cm, which operates at a nominal frequency of 20 KHz. The maximum amplitude and power of the ultrasonicator system were 16 μm and 100 W, respectively. Briefly, 5 g of defatted AOB seed flour was dispersed in 50 mL 0.2 M sodium carbonate buffer (ratio 1:10). The pH of the mixture was adjusted to 11 with 2 M NaOH. and was transferred into a double-walled flow cell (total volume 70 mL, Celbius Ltd., London, UK). Water was circulated through the cell to maintain the extraction temperature constant (50 °C). The horn tip was immersed around 1.5 cm into the solution. Extraction was under three sonication amplitudes that represented sonication intensities, namely low (5 μm), medium (10 μm), and high (15 μm). The ultrasonication extraction was carried out for 15, 30, 45, and 60 min under continuous pulse mode. Following extraction, the mixture was centrifuged at 10,000× *g* for 10 min at 5 °C. Then, supernatants were acidified by adding 2 M HCl until the isoelectric point was reached (pH 4.5, based on preliminary tests on a range of pH values (2–11) and measurement of turbidity). Samples were kept at 4 °C for 2 h and centrifuged at 5000× *g* for 25 min at 4 °C. The precipitates were washed twice with distilled water, centrifuged, and their pH was adjusted to 7 using 2 M NaOH. The neutralised precipitates were freeze-dried for 48 h (Virtis 4K Benchtop, Ipswich, UK) and stored at −20 °C. The protein content of AOB extracts was determined by Bradford protein assay [23]. The protein recovery yield was calculated using the following equation:

(2) $$\mathrm{Recovery}\;\mathrm{yield}\;\left(\%\frac{w}{w}\right)=\frac{\mathrm{protein}\;\mathrm{concentration}\;\mathrm{in}\;\mathrm{the}\;\mathrm{extract}\;}{\mathrm{protein}\;\mathrm{concentration}\;\mathrm{in}\;\mathrm{the}\;\mathrm{defatted}\;\mathrm{seeds}}\times 100$$

### 2.4. Amino Acid Analysis of AOB Protein and Protein Isolates

AOB protein and protein isolates were hydrolysed before derivatisation as described by Chatzifragkou et al. [24]. The hydrolysis process was carried out with 6 N HCl acid with the addition of 0.2% (*w*/*v*) phenol to prevent the halogenation of tyrosine in the samples. Samples were subjected to nitrogen gas to prevent oxidation and kept at 110 °C for 24 h. The EZ-Faast kit (Phenomenex, Macclesfield, UK) for amino acid analysis was used for rapid purification and derivatisation of the hydrolysed samples following the steps described in the user manual. From the hydrolysed samples, 100 μL was neutralised and derivatised. This is based on a solid-phase extraction that binds amino acids and allows the derivatisation in aqueous solution of the amine and carboxylic groups of the amino acids at room temperature [25].

Accurately, 20 µL of hydrolysed samples were pipetted into one glass vial and 200 µL of internal standard (norvaline at 200 µmol/L) were added. A 40 µL resin-packed-sorbent tip was attached to a 1.5 mL syringe. Afterward, the solution was passed through the sorbent tip. This was performed slowly, and the resulting solution was collected in a separate vial. Accurately 200 µL of the washing solution was added. The solution was passed slowly through the same sorbent tip and into the syringe. The liquid from the sorbent bed was drained when the air was let through the sorbent tip. Then, 200 µL of eluting medium was added, which allowed the sorbent to be soaked in it. Afterward, the liquid and sorbent particles were ejected from the tip into the vial. The process of adding the eluting medium was carried out repeatedly until all sorbent particles were removed from the vial. Then, 50 µL of reagent 4 was added, and the liquid was emulsified in the vial using a vortex mixer for about 8 s. This reaction was prolonged for 80 s, and the liquid was emulsified again by performing the vortex mixing a second time for approximately 8 s. Then, 100 µL of Reagent 5 was added to the vial and mixed for 6 s. The liquid was left to stand for 1 min, and the organic layer was transferred to a vial with an insert. Then, this was allowed to evaporate slowly under nitrogen [26]. A total of 100 µL of Reagent 6 was used to dilute the residue, and 2 µL was analysed by GC-MS (Agilent Didcot, UK), according to Elmore et al. [27]. One microliter of the derivatised amino acid solution was injected at 250 °C in split mode at a ratio of 20:1 onto a capillary column (Zebron ZB-AAA) (10 m × 0.25 mm; 0.25 μm film thickness).

### 2.5. Surface Hydrophobicity (Ho)

To determine the surface hydrophobicity of protein isolates, the method of Hayakawa and Nakai [28] was used with modifications, and 1-anilinonaphthalene-8-sulfonic acid (ANS) was used as the hydrophobic probe. A dispersion containing 1 g of protein isolate in 20 mL 0.01 M phosphate buffer (pH 7.0) was prepared. The dispersion was centrifuged at 5000× *g* for 25 min at 10 °C. The concentration of protein in the supernatant was measured using the Bradford method [23]. Dilutions were made from the protein solution with concentrations ranging from 50 to 250 μg/mL. From the dilutions, 4 mL protein solutions at each concentration was taken and transferred into a tube. Then, 50 μL of 8 mM ANS solution (prepared in 0.01 M phosphate buffer, pH 7.0) was added. The fluorescence intensity (FI) of samples (4 mL protein solutions and 50 μL of 8 mM ANS solution) were determined at 385 nm (excitation) and 480 nm (emission) via an Agilent Cary Eclipse fluorescence spectrophotometer using an emission slit of 5 nm. The FI of the ANS solution in 0.01 M phosphate buffer was measured. In addition, the FI of the ANS solution and each diluted sample solution without a probe served as controls. The surface hydrophobicity index was calculated using the initial slope of the FI values of protein dilutions with probe versus the protein concentration as linear regression.

### 2.6. Functional Properties of AOB Protein Isolates

The functional properties of AOB seeds protein isolates were determined, and commercial soy protein isolate served as a benchmark (Protein content: 90% *w*/*w*).

#### 2.6.1. Water Holding Capacity (WHC)

The capacity of AOB protein isolates to retain water was determined according to Deng et al. [29] with some modifications. Briefly, 0.5 g of AOB seed or commercial soy protein isolate was weighed into a pre-weighed centrifuge tube, and 12.5 mL of deionised water was added. The suspension was vortexed thoroughly and left to stand for 2 h. Afterward, the mixture was centrifuged at 5000× *g* for 25 min. The supernatant was discarded, and the weight of the sediment and centrifuge tube was recorded.

The water holding capacity (WHC) was calculated as in the equation below:

(3) $$\mathrm{WHC}\;\left(\frac{g}{g}\right)=\frac{W_{2}-W_{1}}{W_{0}}$$

where W_2_ (g) is the weight of centrifuge tube and precipitated protein after water adsorption; W_1_ (g) is the weight of centrifuge tube and protein sample and W_0_ (g) is the weight of protein samples.

#### 2.6.2. Oil Adsorption Capacity (OAC)

The oil adsorption capacity of the protein isolates was determined according to Deng et al. [29] with some modifications. Briefly, 0.5 g of protein isolate was weighed into a pre-weighed centrifuge tube and 7.5 mL of olive oil (Filippo Berio extra virgin olive oil, Italy) was added. This was vortexed thoroughly and left to stand for 2 h. Afterward, the mixture was centrifuged at 5000× *g* for 20 min. The oil was discarded, and the weight of sediment and centrifuge tube was taken.

The oil adsorption capacity (OAC) was calculated as below:

(4) $$\mathrm{OAC}\;\left(\frac{g}{g}\right)=\frac{W_{2}-W_{1}}{W_{0}}$$

where W_2_ (g) is the weight of the centrifuge tube and precipitated protein after adsorbing oil; W_1_ (g) is the weight of the centrifuge tube and protein sample; and W_0_ (g) is the weight of protein.

#### 2.6.3. Foaming Capacity (FC) and Foam Stability (FS)

The foaming capacity and foaming stability of protein isolates were determined by a modified method [30]. The aqueous dispersion of protein isolates (25 mL) was prepared at 1, 2, 3, 4 and 5% (*w*/*v*). The dispersion was mixed at 12,000× *g* using a high-speed homogeniser (IKA-T18, ULTRA-TURRAX) for 2 min. Mixed solutions were transferred to a measuring cylinder (100 mL), and the volumes were recorded at 0 and 30 min [31]. The FC was expressed as the percentage increase in foam volume at time zero, just after mixing, while FS was expressed as the foam volume, which remained after 30 min. The equations below were used to calculate the foaming capacity (FC) and foam stability (FS),

(5) $$\mathrm{FC}\;\left(\%\right)=\frac{V_{0}}{V_{i}}\times 100$$

(6) $$\mathrm{FS}\;\left(\%\right)=\frac{V_{30}}{V_{0}}\times 100$$

where V_i_ V_0_ and V_30_ (mL) are the foam volumes at initial stage of preparation before homogenisation, 0 min and 30 min, respectively, after homogenisation.

#### 2.6.4. Emulsifying Activity Index (EAI) and Emulsion Stability Index ESI

The Emulsifying Activity Index (EAI) and Emulsion Stability Index (ESI) of protein isolates were determined using the turbidity method as described by Deng et al. [29].

Dispersions of AOB or commercial soy protein isolates were prepared with deionised water within a range of concentrations (1, 2, 3, 4, and 5% *w*/*v*). For each sample, 15 mL of the dispersion was transferred to a tube for homogenisation, and 5 mL of olive oil was added. The mixture was homogenised using a high-speed homogeniser (IKA-T18, ULTRA-TURRAX) at 16,000 rpm for 2 min. Immediately after homogenisation, at time *t*_0_, 30 μL of the emulsion was taken from the bottom of the tube and transferred into a separate tube containing 12 mL of 0.1% *w*/*v* SDS. The absorbance of the diluted emulsion was determined at 500 nm using a spectrophotometer. The emulsion stability Index ESI was determined by measuring the EAI value at 30 min and calculating it as seen in the equations below:

(7) $$\mathrm{EAI}\;\left(m^{2}/g\right)=\frac{2\times 2.303\times \;A0\times \;\mathrm{Dilution}\;\mathrm{factor}}{C\;\times \;\varphi \;\times 10\;}$$

(8) $$\mathrm{ESI}\;\left(\%\right)\;\frac{{\mathrm{EAI}}_{30\min}}{{\mathrm{EAI}}_{10\min}}\times 100$$

where A0 is the absorbance of the diluted emulsion after preparation at 0 min. Dilution factor = 100. C is the concentration of the sample (mg/ mL). φ is the oil volumetric fraction of emulsion (ml/mL). EAI_30min_ is the EAI determined at 30 min, EAI_10min_ is the EAI determined at 10 min, and 10 mm is the path length.

### 2.7. Statistical Analysis

All experiments were carried out in 2 replicates (acid-soluble lignin determination) or 3 replicates (protein extraction, characterisation, and functional properties), and data obtained were presented as mean values of replicates. The standard deviations from the mean were also calculated, and these were performed using Microsoft Excel.

## 3. Results and Discussion

### 3.1. Compositional Analysis of AOB Seeds

The chemical composition of two batches of AOB seeds (AOB 1 and AOB 2) is presented in Table 1.

The moisture content of AOB 1 and AOB 2 were 6.5 and 11% (*w*/*w*), respectively (*p* < 0.0001). This difference in moisture content could be due to seasonal variations and the harvest time of the different batches [32]. Igwenyi et al. [4] also recorded low moisture content in AOB at 5.7% (*w*/*w*). The ash content values of AOB 1 and AOB 2 were not significantly different (*p* = 0.283). Enujiugha [19] and Onyekachi et al. [5] recorded similar ash contents in AOB seeds (2.1 and 2.7% *w*/*w*, respectively). With regards to total lipid content, AOB 1 had a significantly higher lipid content (46% *w*/*w*) than AOB 2 (39% *w*/*w*) (*p* < 0.0001). The lipid content of AOB 1 is in a similar range to values reported previously by Ikhuoria et al. [33] (47% (*w*/*w*) total lipid content) and by Igwenyi et al. [4], who reported 48% *w*/*w* lipid content in AOB. Protein content in AOB seeds was 27% (*w*/*w*), slightly higher than values reported in the literature (22–23%, *w*/*w*) [34,35]. Acid-soluble lignin of AOB seeds was found to be around 3.0% for both batches. Lignin content is higher in most seed hulls and provides rigidity to the plant cell wall by binding with hemicellulose and cellulose, thus forming lignin–carbohydrate complexes [36]. James et al. [37] recorded total lignin content of 7% in AOB seeds, as opposed to 3% of acid-soluble lignin content reported in the current study. With regards to AOB carbohydrate content, it was found to vary slightly between the two AOB seed batches (Table 2). Batch 2 had a higher proportion of cellulose (4.4% *w*/*w*) and hemicellulose (7.1% *w*/*w*) compared to batch 1. Nevertheless, the ratio of cellulose to hemicellulose was approximately 0.6 for both AOB batches. There is scarce information on the carbohydrate content of AOB seeds in the literature; James et al. [37] calculated the cellulose and hemicellulose content of AOB as 44 and 17% (*w*/*w*), respectively. This was calculated as a percentage of the total carbohydrate and not as a fraction of the total seeds’ composition as carried out in this study.

Differences in carbohydrate composition could be a result of variations in growing conditions that entail soil type, climatic conditions such as drought or rainfall, and fertiliser application [38]. The mean temperature in Nigeria in 2016 (26.02 °C) was slightly lower than that in 2017 (26.38 °C) [39]. Since no major differences in macronutrient composition were identified between batches, further experiments were performed using batch 1 AOB seeds as the starting material.

### 3.2. Ultrasound-Assisted Extraction of Defatted Seeds

Ultrasonication was investigated as means of extracting proteins from AOB seeds at pH 11 using sodium carbonate buffer. Therefore, extraction time and ultrasonication intensity were the two parameters under evaluation.

**Figure 1:**
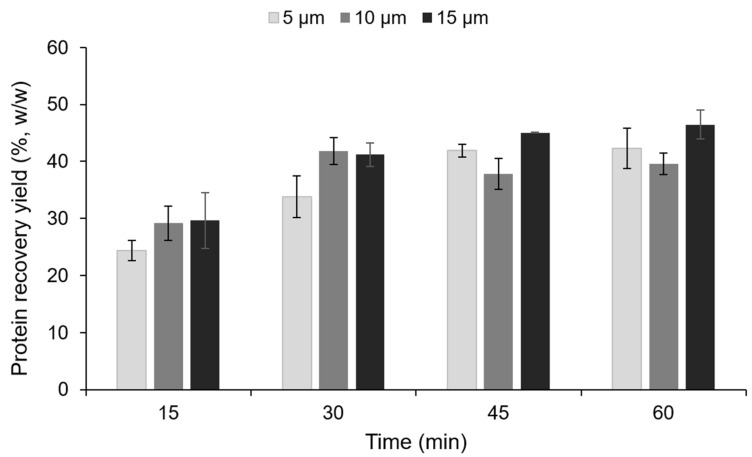
Protein recovery yield (%, *w*/*w*) from defatted AOB seeds by ultrasonication-assisted extraction at 50 °C.

As seen in Figure 1, the duration of extraction worked more favourably for AOB protein extraction, as opposed to extraction intensity. Specifically, the increase in extraction time from 15 min to 60 min led to an almost two-fold increase in the overall protein extraction from 24% to 42% (*w*/*w*), even at low intensity (5 μm). Increasing the intensity of the process at 15 μm did not lead to significant improvement in protein extraction, with the only exception being that of 30 min when a rise in intensity from 5 to 10 μm led to recovery yield improvement by 23% (from 33.8% to 41.8%, *w*/*w*).

During ultrasonication, the generation of cavitation bubbles allows the local disruption of the plant cell wall and the increased penetration of alkaline solvent into the cellular materials, thereby resulting in the release of intracellular products [40]. In addition, the sonication process imparts heat and combined with mechanical effects, promotes the opening of pores in the seeds and causes a higher rate of mass transfer and release of proteins and other intracellular components, which results in higher extraction yields [13,41]. Chittapalo and Noomhorm [42] reported that during the extraction of protein from rice bran, ultrasonication led to a higher yield of protein recovery (about 1.65 times more) compared to that during the alkaline extraction method only. It was also reported that the recovery yield increased with increasing intensity and elapsed time. Dong et al. [22] compared the ultrasound-assisted method for protein extraction with that of the conventional water bath method and reported an increased extraction efficiency of 35% during ultrasonication. In addition, Preece et al. [16] reported an increased yield of 10% in soybean protein extraction using ultrasound within the first min of the extraction process, while Eze et al. [12] recorded an increase in the extraction yield of soybean residue (okara) protein by 2.5-fold at 10 µm and 15 µm.

The cell wall composition of AOB seeds plays a key role in protein extractability. The fact that only 46% of the initial protein was extracted with ultrasonication suggests that for AOB seeds, the ultrasonication process is not selective and apart from proteins, other cell wall materials may also be co-extracted (e.g., structural polysaccharides). Non-selective protein extraction via ultrasonication has been previously reported for legumes in the literature, both for low-power (as in the case of this study) and high-power treatments [43,44].

### 3.3. Amino Acid Profile of Defatted AOB Seed and Protein Isolates

**Figure 2:**
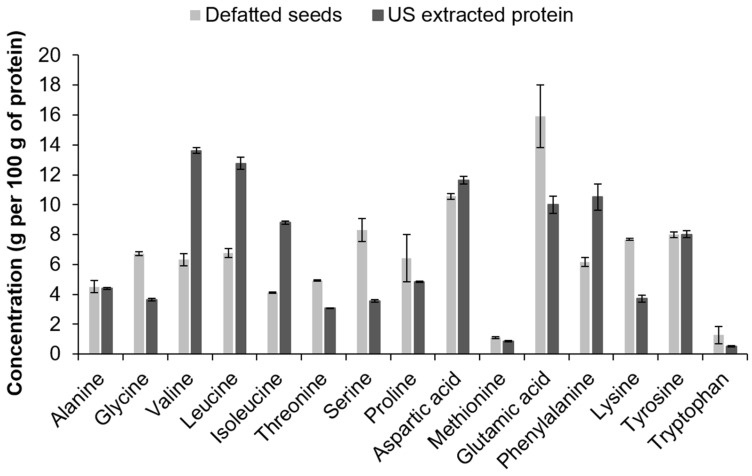
Amino acid profile of African oil bean seed protein and protein isolates extracted by ultrasonication. Extraction conditions: 50 °C, 15 μm, 60 min.

AOB seed protein isolates generated via ultrasonication extraction were analysed for their amino acid content. A comparison between the amino acid profile of the protein extracts and protein in defatted AOB seeds is depicted in Figure 2.

In AOB protein, glutamic acid was the dominant amino acid, which is characteristic of legume proteins [45]. Defatted samples of AOB seeds also had high values of aspartic acid. AOB seeds contained relatively high amounts of essential amino acids. Eight of the essential amino acids were identified, with lysine and valine being the predominant essential amino acids in AOB, whereas the low levels of methionine in AOB seeds agree with literature since it is the limiting essential amino acid in legumes [46].

Worth mentioning is the fact that AOB isolates had a more hydrophobic amino acid profile, as indicated by their content in valine, leucine, and phenylalanine. The hydrophobic/hydrophilic ratio of AOB protein from ultrasonication was higher (2.2) than that of the protein in defatted AOB seeds (1.1). Ultrasonication has been reported to induce changes in the amino acid composition of proteins, and these are mainly attributed to the intensity of the process as well as the type of protein [15]. This was further supported by evaluating the surface hydrophobicity of protein isolates. The surface hydrophobicity index (H_o_) for AOB protein isolates from ultrasonication extraction (15 μm, 60) was 3813, indicating a high proportion of hydrophobic regions being exposed at its surface [29]. Although the total hydrophobic amino acids content can provide an indication of the hydrophobicity of a protein molecule, the surface hydrophobicity is more reliable since it shows the amount of the hydrophobic regions at the surface, which is the main determinant of the surface-active properties of proteins. This can be seen in cases when used as emulsifiers. Proteins interact more significantly with the oil surface if a large number of hydrophobic amino acids are at the surface [47]. The H_o_ of rapeseed protein isolate and Chinese quince seed protein isolate was reported as 1683 and 933, respectively [22,29]. Kingwascharapong et al. [41] recorded higher surface hydrophobicity of locust bay proteins using ultrasonication compared to the typical protein extraction method without ultrasonication. This was attributed to the cavitation effect of ultrasonication, which results in the unfolding of protein molecules wherein hydrophobic domains become more exposed during the process [48,49].

Apart from the amino acid composition, other factors such as temperature, pH, extraction conditions and drying methods also affect protein surface hydrophobicity [29]. Since the temperature has been reported in the literature as a key parameter that influences H_o_, the temperature of extraction of AOB protein isolates could also be a major contribution to their H_o_ levels. Shen and Tang [50] recorded an increase in H_o_ from 2000 to 5600 when soy protein isolates were heated to 75 °C; however, when heated further up to 95 °C, the H_o_ decreased to 2800. This increase in H_o_ with heat depicts the exposure of hydrophobic regions that were initially buried within the protein molecule to the surface. In contrast, the decrease in H_o_ after 75 °C was due to the heat-induced aggregation of thermally unfolded and denatured proteins [50]. Generally, the surface hydrophobicity of AOB seeds was relatively high, which indicates that they could be used as emulsifiers in food products. They are also seen to be in a similar range with soy protein isolate, which served as a benchmark in the current study.

### 3.4. Functional Properties of AOB Protein Isolates

A number of functional properties of AOB seeds protein isolates from batch 2 were evaluated, and commercial soy protein isolate was used as a benchmark. Initially, water holding capacity (WHC) and oil adsorption capacity (OAC) were investigated as useful parameters that often determine the suitability of protein isolates as ingredients in food formulations. The results are presented in Table 3.

In terms of WHC, soy protein isolates had the capacity to bind more than twice the quantity of water per g of protein isolate than AOB (8 g/g compared to 4.2 g/g). This can be associated with the type and number of polar groups in the protein polypeptide chain. Soy protein isolates have large numbers of polar hydrophilic groups, such as serine and threonine [51], while AOB protein isolates have very low amounts of these amino acids. The water absorption capacity of proteins is determined by certain factors, including their conformation, shape, size and the hydrophilic-to-hydrophobic ratio of amino acids [52]. With regard to OAC, soy protein exhibited a higher value (2.2 g/g) than AOB seed protein, at 1.8 g/g. OAC influences food texture, mouthfeel, and flavour retention. The oil adsorption capacity of proteins has been shown to be influenced by the protein–lipid interactions as well as the spacing of the lipid phase [47]. Since nonpolar side chains of protein molecules are the core sites of lipid–protein interactions, the relatively high OAC values of soy can be attributed to the number of nonpolar sites in soy protein isolates. The secondary structure of proteins also has a significant effect on their ability to adsorb oils.

Another property under investigation was that of foaming capacity. Generally, proteins are suitable surface-active agents for stabilising a gaseous dispersed phase in food products. For foams to be formed, a large interfacial area is required to enhance the inclusion of air in the liquid phase and the build-up of an interfacial film that is resistant to agents that can collapse the bubbles. The ability of proteins to form and stabilise foams is dependent on the degree of hydrophobicity, the distribution of charges and the net charge [53]. The FC was above 200% for both protein isolates (Table 4). AOB had a high FC at 2% *w*/*v* (258%), indicating the likelihood of inherent structural features such as the presence of amphipathic molecules that can produce sufficient interaction of protein molecules at an air–water interface; thus, yielding a continuous interfacial film that envelopes the nascent bubbles [54]. AOB protein isolates had 34 g/100 g of hydrophobic and 15 g/100 g of hydrophilic amino acids. This resulted in a hydrophobic/hydrophilic ratio of 2.2 for AOB seed protein isolates. The relatively high hydrophobicity of AOB protein isolates likely contributed to their foaming capacity. These results are in a similar range with FC values in the literature for commercial soy protein isolates (235%) and sunflower protein isolates (230%) [55].

Different factors can contribute to the FC of protein isolates. For instance, pH value has been considered a significant factor in the FC of protein isolates since it determines the solubility of proteins. It is possible that the pH of the solvent (pH 12) contributed to the high FC and FS values of AOB and soy protein isolates. Although the presence of amphipathic molecules is required in foams, higher solubility improves protein–water interactions as well as leads to more protein unfolding [29]. In terms of foaming stability, as seen in Table 4, the foams had high stability of over 96% at all concentrations studied except the foams of AOB protein isolates prepared at 1% *w*/*v*. The foaming stability of AOB was higher than values reported in the literature for other seed proteins, such as sunflower protein isolates (36%) and local beans of Turkey (81%) [31,56]. Generally, the results indicate that the interfacial film formed by AOB and soy protein isolates possesses desirable characteristics such as having suitable mechanical strength as well as being impermeable and flexible.

The emulsifying properties of AOB and commercial soy protein isolates were tested at pH 12, where the solubility of the protein isolates was highest compared to other pH levels (data not shown). The Mie theory for light scattering shows that there is a simple relationship between turbidity and the interfacial area of an emulsion. Therefore, the capacity of proteins to serve as emulsifiers and stabilise emulsions formed is proportional to the interfacial area that can be coated by the protein [57,58].

Soy had higher EAI (85 m^2^/g) than AOB seed protein isolates at 51 m^2^/g (Table 5). These values serve as indicators of the ability of AOB protein isolates to facilitate the formation of emulsions [54]. EAI values decreased consistently with an increase in concentration for AOB and soy. This is likely because the adsorption of proteins at the oil/water interface is diffusion controlled, and at low protein concentrations, protein molecules would have to spread over the surface prior to adsorption. However, as concentration increases, the activation energy barrier formed hinders the diffusion-dependent process of adsorption. Hence the emulsifying activity decreases as the concentration increases [52]. The rate at which protein molecules diffuse towards the oil/water interface is a key determinant factor for the quantity of protein that gets adsorbed to the interface when an emulsion is formed. As such, factors that decrease the rate of diffusion of these protein molecules, such as an increase in protein concentration, would consequently reduce the amount of protein adsorbed, thereby reducing the emulsifying ability index [54]. This can be supported by the fact that EAI values were in inverse order with the protein content for all protein isolates (Table 5). This trend was also reported by Sze-Tao and Sathe [59], who observed a decrease in EAI of walnut protein isolates with increasing protein concentration. In addition, it was observed in previous studies that the emulsifying ability of walnut protein concentrate (with lower protein content) was higher than the emulsifying ability of walnut protein isolate, which had a higher protein content [52].

As shown in Table 5, emulsions containing soy isolates exhibited high stability with an ESI value of 104% at 1% *w*/*v*, and these ESI values were approximately the same at all concentrations evaluated. The ESI values were initially lower in AOB protein isolates at 78% for 1% *w*/*v* and exhibited a constant increase as the concentration of the solution increased (Table 5). Although increases in protein concentration did not improve the EAI of the protein isolates, in AOB protein isolates, it led to an increase in the stability of the emulsion. This could be because, as the protein content increased, there were more protein molecules present, thus forming a substantial interaction of protein molecules due to high hydrophobic interactions at the protein surface, resulting in a strong oil–water interface, which characterises stable films [60].

Proteins have the ability to stabilise emulsions due to their capacity to prevent the emulsified droplets from uniting and forming larger droplets, which results in phase separation. Emulsion stability can be estimated by the degree to which proteins unfold, which mainly determines their tendency to form an interfacial film around the emulsion droplets. As the proteins unfold at the oil–water interface, the number of contact points per molecule increases with the interface, which reduces the likelihood of the desorption of the molecular segments [60]. This process of unfolding at the oil/water interface is largely determined by the stability of the protein tertiary structure and some external factors such as temperature, presence of denaturants, and pH. The lower ESI values of AOB protein isolates were not expected because the surface hydrophobicity values for AOB protein isolates were higher than that of soy. However, surface hydrophobicity may not be the only factor that determines ESI. Some hydrophobic protein molecules may adsorb to the oil/water interface but may not have sufficient flexibility for reorientation. Thus, the flexibility of protein segments also contributes to the stability of emulsions. It has been shown that heat denaturation is beneficial for the increase in emulsifying stability. However, this occurs up to a certain degree beyond which the proteins can get denatured to a point where they cannot be used. This partly explains why AOB protein isolates had lower ESI [60].

## 4. Conclusions

AOB seeds are rich in protein and lipids and contain minerals in significant quantities, as indicated by the total ash content. The presence of lignin in the seeds provides an explanation for their rigid structure. The chemical composition of the seeds shows that they can be exploited for diverse purposes in the food industry.

Ultrasonication favoured protein extraction and influenced the properties of AOB protein. Further work should be carried out to assess the scalability of the ultrasonication process, as the evidence provided in this study is based on a lab scale. Desirable changes to the functional properties of AOB protein isolates were also noted, such as the increase in the hydrophobic amino acids, which led to the relatively high emulsifying properties. These findings serve as a basis for future investigation of AOB protein isolates as ingredients within food matrices, such as plant-based emulsifiers for mayonnaise and salad dressings, as well as in foams to impart body, smoothness, and lightness to foods. This study could help increase the awareness of AOB protein applications and consequently improve AOB seed utilisation, leading to economic benefits through the provision of an alternative protein ingredient and contributing to food security and sustainability in tropical Sub-Saharan regions.

## Figures and Tables

**Table 1:** Compositional analysis of two batches of African oil bean (AOB) seeds (in %, *w*/*w*).

Sample	Moisture	Ash	Lipid	Protein	Carbohydrates	Acid-Soluble Lignin	Total
AOB1	6.8 ± 0.3 ^b^	2.2 ± 0.2 ^c^	46 ± 2 ^b^	27 ± 1 ^b^	8.8 ± 0.3 ^b^	2.9 ± 0.1	93 ± 2
AOB2	11 ± 1 ^a^	2.4 ± 0.1 ^c^	38 ± 1 ^c^	27 ± 1 ^b^	11 ± 1 ^a^	3.0 ± 0.1	94 ± 1

Values are expressed as means of three replicates ± standard deviation. ^a–c^: Represent the Tukey test for multiple comparisons. Mean values with the same letter in rows are not significantly different (*p* ≤ 0.05). AOB1: AOB seeds from batch 1; AOB2: AOB seeds from batch 2.

**Table 2:** Carbohydrate polymers in African oil bean (AOB) seeds.

Carbohydrate	AOB 1 (% *w*/*w*)	AOB 2 (% *w*/*w*)
Cellulose	3.2 ± 0.1 ^c^	4.4 ± 0.2 ^a^
Hemicellulose	5.6 ± 0.1 ^b^	7.1 ± 0.1 ^a^
Total	8.8 ± 0.3 ^b^	11 ± 1 ^a^

Values are expressed as means of three replicates ± standard deviation. ^a–c^: Represent the Tukey test for multiple comparisons. Mean values with the same letter in rows are not significantly different (*p* ≤ 0.05). AOB1: AOB seeds from batch 1; AOB2: AOB seeds from batch 2.

**Table 3:** Water holding capacity (WHC) and oil adsorption capacity (OAC) of commercial soy and AOB protein isolates.

Sample	WHC (g/g)	OAC (g/g)
Commercial soy	8.0 ± 0.8	2.2 ± 0.2
AOB	4.2 ± 0.1	1.8 ± 0.1

Values are expressed as means of three replicates ± standard deviation.

**Table 4:** Foaming capacity (% *v*/*v*) of commercial soy protein isolates and AOB seeds protein isolates.

Concentration (% *w*/*v*)	Foaming Capacity (% *v*/*v*)		Foaming Stability (% *v*/*v*)	
	AOB	Commercial Soy	AOB	Commercial Soy
1	231 ± 2 ^a^	223 ± 8 ^a^	73 ± 7 ^b^	100 ± 1 ^a^
2	258 ± 1 ^a^	236 ± 10 ^a^	96 ± 2 ^a^	99 ± 0 ^a^
3	254 ± 6 ^a^	227 ± 13 ^a^	98 ± 1 ^a^	98 ± 0 ^a^
4	246 ± 5 ^a^	219 ± 5 ^a^	97 ± 2 ^a^	98 ± 0 ^a^
5	242 ± 3 ^a^	226 ± 9 ^a^	97 ± 1 ^a^	98 ± 1 ^a^
Mean	**246 ± 11** **^ a ^**	**226 ± 7** **^ b ^**	**92 ± 11** **^ b ^**	**99 ± 1** **^ a ^**

Values are expressed as means of three replicates ± standard deviation. Letters: Represent the Tukey test for multiple comparisons between soy and AOB and among the concentrations (interaction between protein isolates and their concentration). Mean values with the same letter in rows are not significantly different (*p* ≤ 0.05). Values in bold are means of the main effects of AOB and commercial soy protein isolates.

**Table 5:** Emulsifying ability index (EAI) m^2^/g and emulsifying stability index (ESI) (%) of commercial soy and AOB seeds protein isolates.

Conc. (% *w*/*v*)	EAI (m^2^/g)		ESI (m^2^/g)	
	AOB	Soy	AOB	Soy
1	51 ± 3 ^b^	85 ± 5 ^a^	78 ± 9 ^c^	104 ± 4 ^ab^
2	31 ± 1 ^c^	47 ± 1 ^b^	88 ± 8 ^bc^	103 ± 2 ^a^
3	22 ± 1 ^ef^	28 ± 1 ^cd^	89 ± 4 ^bc^	106 ± 2 ^a^
4	17 ± 1 ^fg^	25 ± 2 ^cde^	97 ± 1 ^ab^	102 ± 7 ^ab^
5	14 ± 1 ^g^	25 ± 1 ^def^	101 ± 4 ^ab^	103 ± 1 ^a^
Means	**27 ± 15 ^b^**	**41 ± 26 ^a^**	**91 ± 9 ^b^**	**103 ± 2 ^a^**

Values are expressed as means of three replicates ± standard deviation. Letters: Represent the Tukey test for multiple comparisons between soy and AOB and among the concentrations (interaction between protein isolates and their concentration). Mean values with the same letter in rows are not significantly different (*p* ≤ 0.05). Values in bold are means of the main effects of AOB and commercial soy protein isolates.

## Data Availability

Data sharing not applicable.
